# Autism Spectrum Disorder: From Experimental Models to Probiotic Application with a Special Focus on *Lactiplantibacillus plantarum*

**DOI:** 10.3390/nu17152470

**Published:** 2025-07-29

**Authors:** Giusi Sabatini, Ilenia Boccadoro, Roberta Prete, Natalia Battista, Aldo Corsetti

**Affiliations:** Department of Bioscience and Technology for Food, Agriculture and Environment, University of Teramo, 64100 Teramo, Italy; gsabatini@unite.it (G.S.); iboccadoro@unite.it (I.B.); nbattista@unite.it (N.B.); acorsetti@unite.it (A.C.)

**Keywords:** autism spectrum disorder, probiotics, *Lactiplantibacillus plantarum*, multifactorial biomarkers, experimental models, clinical trials

## Abstract

Background/Objectives: Autism spectrum disorder (ASD) encompasses several neurodevelopmental disorders, whose onset is correlated to genetic and environmental factors. Although the etiopathogenesis is not entirely clear, the involvement of inflammatory processes, the endocannabinoid system, and alterations in the permeability and composition of the intestinal microbiota are known to occur. Methods: This review systematically explores the literature available to date on the most widely used murine models for the study of ASD, the main biomarkers investigated for the diagnosis of ASD, and the therapeutic potential of probiotics, with a particular focus on the use of strains of *Lactiplantibacillus* (*Lpb.*) *plantarum* in in vivo models and clinical trials for ASD. Results: Several studies have demonstrated that targeting multifactorial biomarkers in animal models and patients contributes to a more comprehensive understanding of the complex mechanisms underlying ASD. Moreover, accumulating evidence supports the beneficial effect of probiotics, including *Lpb. plantarum*, as a promising alternative therapeutic strategy, capable of modulating gut–brain axis communication. Conclusions: Probiotic supplementation, particularly with selected *Lpb. plantarum* strains, is emerging as a potential complementary approach for ameliorating ASD-related gastrointestinal and behavioral symptoms. However, further large-scale clinical studies are essential to validate their efficacy and determine optimal treatment protocols and dietary strategies.

## 1. Introduction

In recent decades, the scientific community has devoted considerable attention to the study of neurodevelopmental disorders, such autism spectrum disorder (ASD). Traditionally, research has focused on understanding the neurological and behavioral components of these conditions. However, emerging evidence increasingly underscores their multifactorial etiology and complex pathophysiology, prompting a paradigm shift in investigative approaches and conceptual frameworks.

This review is aimed at providing an extensive review of preclinical and clinical studies related to the possibility of using probiotics in the treatment of ASD. The various multiple factors and biomarkers involved in the ASD pathophysiology will be discussed, along with a special focus on the available experimental animal models currently applied for studying ASD, for which limited information is provided. Preclinical and clinical studies involving the use of *Lactiplantibacillus* (*Lpb.*) *plantarum* strains as probiotics in individuals with ASD will be reported, offering a promising dietary strategy to ameliorate ASD symptoms.

## 2. Autism Spectrum Disorder

ASD is a group of heterogeneous neurodevelopmental conditions [1], characterized by persistent deficits in the ability to initiate and to sustain reciprocal social interaction and social communication, and by a range of restricted, repetitive, and inflexible patterns of behavior, interests, or activities that are clearly atypical or excessive for the individual’s age and sociocultural context, according to the 11th revision of the International Classification of Diseases (ICD-11) [2]. Individuals with ASD often exhibit language and speech impairments, intellectual disability, learning difficulties, motor dysfunctions, and deficits in social communication and interaction [3]. The expression and severity of ASD symptoms vary widely between individuals and involve a broad range of behavioral patterns, with different intensities [4]. In addition, ASD is frequently associated with other clinical features, including gastrointestinal (GI) problems (up to 70% of cases), motor deficits (79%), sleep disturbances (50–80%), and intellectual disability (45%) [5]. Moreover, it should be recalled that ASD is often accompanied by comorbidities, including epilepsy, depression, anxiety, and attention-deficit hyperactivity disorder (ADHD) [6].

International epidemiological studies report a general increase in ASD prevalence. A recent global meta-analysis involving over 21 million children estimated the prevalence of ASD at 0.77%, with a higher rate in males (1.14%). The data, drawn from 66 studies conducted primarily in Europe, Asia, and the Americas, revealed considerable regional variation, with Australia reporting the highest prevalence, highlighting it as a key area for public health attention [7]. In Italy, it is estimated that 13.4 per 1000 children are diagnosed with ASD, with a male-to-female ratio of 4.4:1 [8]. Although ASD diagnoses are increasing worldwide, there is currently no FDA-approved pharmacological treatment to alleviate the core symptoms of ASD [9], nor have any been approved by the Italian authorities.

Despite numerous studies aiming to investigate the etiology of ASD, the exact cause remains unclear. It is suspected that both genetic and environmental factors influence the ASD phenotype [10]. In fact, ASD shows strong genetic components, with heritability estimates ranging from 60% to over 80% [11]. Genetic and chromosomal abnormalities, such as fragile X syndrome (FXS), Tuberous Sclerosis Complex (TSC), and alterations in chromosomal regions 2q, 7q, 15q, and 16p have been identified in 35–40% of ASD cases [12]. The genetic basis of ASD is highly heterogeneous, and only a few genes have been well-characterized, including the Src Homology 3 (SH3) domain, SHANK3 (SH3 and multiple ankyrin repeat domains protein 3), contactin-associated protein-like 2 (CNTNAP2), and, more recently, chromodomain helicase DNA-binding protein 8 (CHD8) [13].

**Figure 1:**
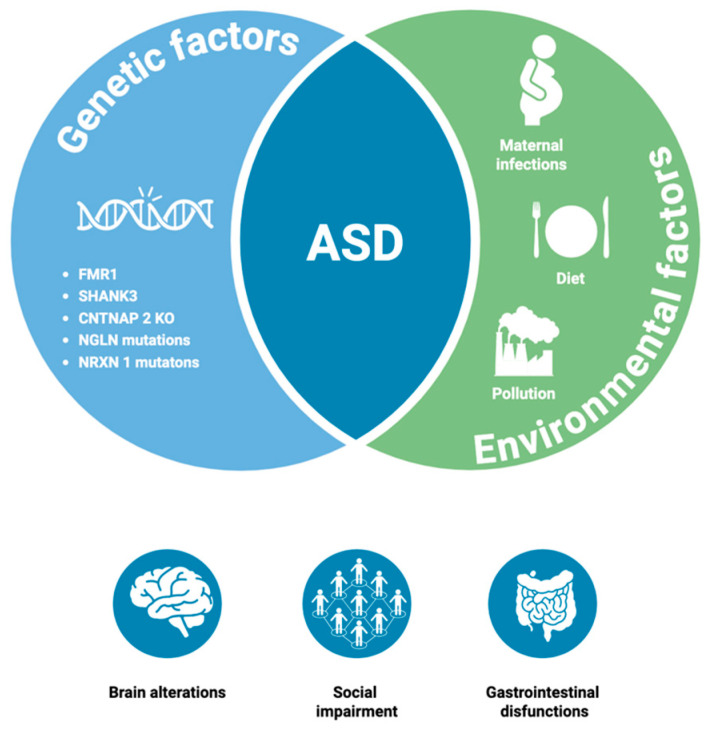
Overview of the main factors characterizing ASD. The figure illustrates the combination of genetic and environmental factors underlying autism spectrum disorder (ASD). Specifically, the genetic factors include fragile X messenger ribonucleoprotein 1 (FMR1) gene, SH3 and multiple ankyrin repeat domains 3 (SHANK3), contactin-associated protein-like 2 (CNTNAP2) knockout, Neuroligin (NLGN) mutations, and Neurexin (NRXN1) mutations. The environmental factors shown include maternal infections, dietary influences, and exposure to pollution. At the bottom of the figure, three of the main symptoms associated with ASD are presented: brain alterations, social impairment, and gastrointestinal dysfunction (Created in BioRender. https://BioRender.com/4edg0mn, URL accessed on 15 June 2025).

Air pollution, pesticide exposure, various maternal medical conditions, including diabetes, dietary factors, such as differences in maternal nutrients and antibiotic use during pregnancy, have been included among the potential environmental contributors [10,14]. Therefore, ASD is currently considered to be a complex disorder, with a multifactorial etiopathogenesis, likely resulting from the combined effects of genetic, epigenetic, and environmental risk factors [1]. Figure 1 presents a schematic summary of the main risk factors characterizing ASD.

## 3. In Vivo Experimental Models of ASD

An in-depth investigation of the genetic and environmental mechanisms underlying ASD has led to the development of research models, including animal models, which currently enable the advancement of diagnostic methods, therapeutic strategies, and preventive approaches for this disorder. Several rare Mendelian syndromes, as well as structural chromosomal variations, are recognized as high-risk factors for ASD. Moreover, genome-wide studies have identified numerous risk loci, also shared with other neurodevelopmental disorders, including schizophrenia and intellectual disability. Copy number variants (CNVs) and de novo single nucleotide mutations also contribute significantly to ASD etiology [15].

Prenatal environmental factors can also lead to malfunctions in the immune system during early development and increase ASD risk [14]. Given the limited access to human samples, the use of animal models is essential both to understand the neurobiological aspects of ASD and to test potential therapies; in particular, due to their behavioral and genetic correspondence to humans, mice are one of the most used models [16], together with zebrafish (*Danio rerio*) that, thanks to them having a similarity to human genes of approximately 70%, along with their transparent body and high reproductive rate, is also a suitable model for high-throughput studies [17].

An ideal model should have: face validity, construct validity, and predictive validity [18].

As of April 2025, the database, SFARI Gene, includes 268 genetic, 45 environmental and 8 idiopathic mouse models, and 1230 human genes that are potentially implicated in the development of ASD (https://gene.sfari.org/database/human-gene/, URL accessed on 22 April 2025).

### 3.1. Genetics Models

Genetic susceptibility to ASD involves hundreds of genes that affect synaptic transmission, neuronal excitability, postsynaptic density protein regulation, cell adhesion, and chromatin remodeling during neurogenesis [19]. These murine models are typically classified into knockout (KO)/knock-in (KI) models, transgenic models, and natural or inbred strains.

The most widely used genetic models are fragile X messenger ribonucleoprotein 1 (FMR1), SHANK 3, CNTNAP 2, Neuroligins (NLGNs), and Neurexins (NRXNs).

SHANK 3 (proline-rich synapse-associated protein 2 (ProSAP2)) encodes a postsynaptic scaffolding protein that is critical for excitatory synapse function. Several *Shank3* mutant mice were generated to elucidate the role of *Shank3* in synapse function and brain development; the KO model is largely used in experimental research, since it displays severe impairments in behavioral and synaptic traits, such as social deficits, anxiety-like behaviors, repetitive grooming, and synaptic abnormalities [20]. In Shank3Δ11^−/−^ mice, exon 11 is deleted, which is involved in synaptic function and neuronal maturation [21]. These Shank3 KO mice show stereotyped behavior, impaired social interaction, and motor dysfunction [20].

Fragile X syndrome (FXS), one of the most common genetic causes of ASD, results from mutations in the X-linked FMR1 gene. The 5’ untranslated region of FMR1 contains a CGG triplet repeat, whose abnormal expansion (>200 repeats) can cause the epigenetic silencing of FMR1 and, thus, a loss of FMR1-encoded fragile X messenger ribonucleoprotein 1 (FMRP). FXS patients exhibit a series of cognitive and behavioral deficits, such as intellectual disability, language and sensor impairment, social defects, and stereotyped and repetitive behavior. Loss of the FMR1 gene in mice leads to an overlapping symptomatology with humans [22].

CNTNAP2 KO mice are susceptible to disruptive mutations that carry heterozygous variants and present intellectual disability, seizures, autistic traits, and language impairments [23].CNTNAP2 encodes CASPR2, which is involved in mediating cell–cell communication and forming synaptic bridges that regulate synaptic strength and plasticity, and influences synaptic transmission and dendritic branching. CNTNAP2 is essential for proper cortical development and brain function, and its homozygous loss-of-function mutations are linked to an ASD characterized by cortical malformations, epilepsy, regression in language, and cognitive delay [24]. CNTNAP2 KO mouse models exhibit hyperactivity, spontaneous seizures, altered sleep patterns, impaired sensory processing, and deficits in spatial discrimination [23].

NLGNs are postsynaptic proteins that promote synapse formation and maturation through interactions with presynaptic NRXNs [25]. In humans, five NLGNs isoforms are encoded by NLGN1, NLGN2, NLGN3, NLGN4X, and NLGN4Y. Nlgn3 R451C KI mice exhibit reduced NLGN3 surface expression, impaired social interactions, and increased repetitive actions [26]. Interestingly, these phenotypes are absent in Nlgn3 KO mice, suggesting a gain-of-function mechanism [25]. NRXN1 mutations have been linked to schizophrenia and intellectual disabilities. Deletion models targeting α-Nrxn isoforms show disrupted neurotransmitter release, without major alterations in synapse structure. While Nrxn-1α mutants retain normal sociability, deficits in social memory tasks have been observed [27].

In addition to the genetic models discussed above, several other models have been widely employed in ASD research. Notable examples include MECP2 [28], CHD8 [29] [30], PTEN [31], TSC1 [32], UBE3A [33], and TBR1 [34], which have contributed significantly to the understanding of the molecular and behavioral phenotypes associated with the disorder.

### 3.2. Environmental Models

Environmental factors, such as environmental pollution, chemical exposure, and viral infection, are significant pathogenic contributors to the development of ASD in terms of oxidative stress and impairment of the methylation process [35]. Among these agents, valproic acid (VPA) and polyinosinic–polycytidylic acid (Poly I:C) and lipopolysaccharide (LPS) are used to generate environmentally induced animal models of ASD.

In utero VPA exposure has been linked to an increased risk of ASD, with evidence of reduced sociability, communication deficits, repetitive behaviors, impaired prepulse inhibition, altered pain sensitivity, heightened anxiety, and hyperactivity [36], and also teratogenic effects, such as neural tube defects, cardiovascular and limb anomalies, and neurodevelopmental delays [18]. Neuroanatomical and cellular alterations, including an imbalance in cortical excitation/inhibition, with increased glutamatergic and decreased GABAergic signaling, have also been reported [37]. Additionally, VPA-exposed rodents display immune and gut microbiota changes, resembling those observed in individuals with ASD.

Maternal obesity and a high-fat diet (MHFD) during pregnancy have been associated with an increased risk of ASD in offspring. MHFD induces shifts in maternal and offspring gut microbiota, which can negatively impact neurodevelopment and social behavior [38]. For instance, MHFD significantly impairs the intestinal mucus barrier in offspring, contributing to increased gut permeability and systemic inflammation [39]. Offspring born to high-fat diet mothers also exhibit elevated anxiety-like behaviors, although conditioned fear responses and exploratory behavior may remain intact.

At the neurochemical level, MHFD has been linked to increased levels of the brain-derived neurotrophic factor (BDNF) in the dorsal hippocampus, as well as the upregulation of the GABA_A α2 subunit and 5-HT1A receptors in the ventral hippocampus, suggesting region-specific alterations in synaptic signaling. Additionally, MHFD exposure leads to cognitive deficits and altered sensitivity to *N*-methyl-D-aspartate (NMDA) receptor antagonists in adult offspring, with effects persisting into the second and third generations in a sex-specific manner.

These findings suggest that maternal overnutrition not only increases the risk of obesity in subsequent generations, but also contributes to long-term impairments in learning and memory, and may accelerate pathological brain aging independent of obesity itself [40].

Building on this evidence, germ-free (GF) mouse models have further elucidated the role of the gut microbiota in mediating the neurodevelopmental effects of early-life environmental factors, such as MHFD. GF models have become a valuable tool for investigating the role of the gut microbiota in ASD [41]. Indeed, by eliminating all microbial exposure, GF mice provide a controlled system to assess the causal effects of microbiota on neurodevelopment and behavior. Notably, the colonization of GF mice with fecal microbiota from individuals with ASD induces social deficits and repetitive behaviors, mimicking core ASD phenotypes [42]. These models have also revealed microbiota-dependent alterations in brain gene expression, neurotransmission, and immune responses, supporting the hypothesis of microbiota–gut–brain axis (MGBA) involvement in ASD pathophysiology [42].

While maternal immunoglobulin G (IgG) provides passive immunity to the fetus, it can also transmit pathogenic antibodies capable of causing congenital developmental disorders, particularly in the presence of autoimmune conditions. In rodent models, maternal immune activation (MIA) is typically induced through gestational exposure to immune stimulants, such as poly I:C or LPS, which mimic viral or bacterial infections, respectively [24,43]. MIA leads to heightened levels of pro-inflammatory cytokines, notably IL-6 and IL-17α, disrupting fetal brain development [44].

Behaviorally, MIA-exposed offspring display reduced exploration, impaired motor skills and sociability, increased anxiety, sensory dysfunctions, and stereotypies, mirroring core features of ASD [45]. MIA has been associated with gut microbiota dysbiosis, compromised intestinal barrier integrity, and increased bacterial families, such as *Lachnospiraceae*, *Porphyromonadaceae*, and *Prevotellaceae* [46]. In addition to MIA models, several other environmentally induced models of ASD have been developed, including those based on recombinant interleukin-6 (rIL-6) [47], prenatal exposure to fluoxetine [48], and various stress-related paradigms [49], such as prenatal stress [50].

### 3.3. Idiopathic Models

ASD pathogenesis results from complex multifactorial interactions, and idiopathic inbred rodent models have emerged as valuable tools to better replicate core behaviors and uncover novel genetic risk factors. The inbred line B Black and tan brachyury (BTBR) T + Itpr3tf/J mouse model is characterized by reduced gray matter volumes in the ventral tegmental area, cingulate gyrus, lateral thalamus, posterior thalamus, occipital and parietal cortices, and subcortex, but it is increased in the olfactory bulb, medial prefrontal and insular cortices, amygdala, and dorsal hippocampus [51].

These mice show marked deficits in sociability, repetitive behaviors, and abnormalities in vocal communication [52]. The inbred line BALB/cByJ mouse model displays marked impairments in social behavior and an increase in stereotypic activities, with a reduction in the corpus callosum volume in the mice. BALB/c mice show significant social deficits, diminished ultrasonic vocalizations, heightened anxiety-like behaviors, and elevated repetitive actions, including excessive grooming. However, BALB/cByJ mice require an appropriate control group and do not fully replicate the complete clinical spectrum of ASD phenotypes [53].

Table 1 provides a comprehensive summary of the genetic, environmental, and idiopathic models outlined above and reported in the last ten years.

## 4. Multifactorial Biomarkers in ASD

Despite its increasing prevalence, the biological mechanisms underlying ASD remain only partially understood, and no definitive biomarkers are currently available for diagnosis or prognosis. Given the multifactorial nature of ASD, research is increasingly focusing on the identification of biological indicators that reflect the convergence of multiple pathophysiological processes. These include different alterations, embracing the endocannabinoid system (ECS), intestinal permeability, immune and inflammatory mediators, as well as intestinal microbiota.

**Figure 2:**
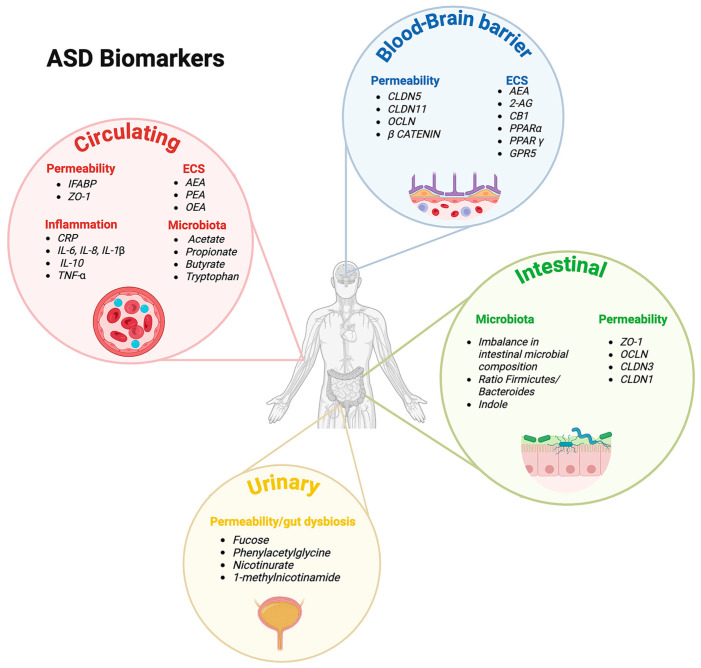
Schematic overview of the main biomarkers associated with ASD (created using BioRender.com). The figure summarizes the main biomarkers associated with ASD, categorized into circulating, blood–brain barrier, intestinal, and urinary biomarkers. Circulating biomarkers are further classified into permeability markers, including intestinal fatty acid-binding protein (I-FABP) and zonulin-1 (zo-1); endocannabinoid system (ECS) markers, including anandamide (AEA), palmitoylethanolamide (PEA), and oleoylethanolamide (OEA); inflammatory makers, such as C-reactive protein (CRP), interleukin (IL)-6, IL-8, IL-1β, IL-10, and tumor necrosis factor-alpha (TNF-α); microbiota-related metabolites, including acetate, propionate, butyrate, and tryptophan. Blood–brain barrier biomarkers include permeability-related proteins, such as claudin-5 (CLDN5), claudin-11 (CLDN11), occludin (OCLN), and β-catenin; ECS components, including AEA, 2-arachidonoylglycerol (2-AG), cannabinoid receptor type 1 (CB1), peroxisome proliferator-activated receptor alpha (PPAR-α), PPAR-gamma (PPAR-γ), and G protein-coupled receptor 55 (GPR55). Intestinal biomarkers are divided into microbiota markers, such as an imbalance in intestinal microbial composition, Firmicutes/Bacteroidetes ratio, and indole; and permeability markers, including ZO-1, OCLN, claudin-3 (CLDN3), and claudin-1 (CLDN1). Urinary biomarkers include markers of permeability and gut dysbiosis, such as fucose, phenylacetylglycine, nicotinurate, and 1-methylnicotinamide (Created in BioRender) https://BioRender.com/4tucvca, URL accessed on 15 June 2025).

Such biomarkers may not only improve diagnostic accuracy and early detection, but also aid in stratifying patients into more biologically homogeneous subgroups. Indeed, this may facilitate the development of targeted therapeutic approaches and personalized interventions. Understanding and validating these complex biomarker networks is essential to advancing precision medicine in ASD. A schematic overview of the main biomarkers associated with ASD is shown in Figure 2.

### 4.1. ECS and ASD

ECS is a complex lipid signaling network involved in the regulation of numerous physiological processes, comprising neurodevelopment, inflammation, and emotional behavior. The discovery of Δ9-tetrahydrocannabinol (Δ9-THC), the psychoactive component of *Cannabis sativa* L., led to the identification of endogenous cannabinoids (endocannabinoids, eCBs), such as anandamide (AEA) and 2-arachidonoylglycerol (2-AG). These ligands act mainly through cannabinoid receptors type 1 (CB1) and 2 (CB2), both G protein-coupled receptors, and are regulated by specific metabolic enzymes. The ECS acts as a homeostatic regulator and has been implicated in various pathological conditions, including ASD [103,104]. Increasing evidence suggests that ECS dysregulation occurs in ASD [105,106].

Preclinical studies in ASD animal models, such as Fmr1-Δexon 8 rats, have revealed region-specific alterations in endocannabinoid tone, such as decreased AEA levels in the hippocampus and elevated 2-AG levels in the amygdala, along with the dysregulated expression of ECS enzymes. Pharmacological interventions targeting these alterations, through the inhibition of AEA degradation (URB597) or CB1 receptor blockade (SR141716A), led to improvements in memory and social behavior, pointing to a causal role of ECS imbalance in ASD-like phenotypes [104].

Other findings show that the increased AEA levels induced by FAAH inhibition improve social impairments through CB1R modulation, while PPARα, PPARγ, and GPR55 expression in the frontal cortex and hippocampus were reduced in ASD rodent models [107]. In line with the involvement of PPARα in ASD pathophysiology, recent studies have demonstrated that the chronic administration of fenofibrate (FBR), a PPARα agonist, from weaning to young adulthood, ameliorated social deficits and repetitive behaviors in VPA male and female rats. FBR treatment also normalized the altered excitatory/inhibitory synaptic balance in the striatum and rescued the blunted dopamine D1 receptor signaling in the nucleus accumbens shell, although this latter effect was sex specific, being evident only in males. These findings further support the therapeutic potential of PPARα activation in mitigating core ASD-like behaviors and neurochemical alterations [108].

Human studies also support ECS involvement in ASD. The reduced expression of CB1 receptors has been observed in post-mortem brain tissue from autistic individuals, while peripheral blood mononuclear cells from ASD patients exhibit increased CB2 gene expression [109]. Furthermore, lower circulating levels of AEA, palmitoylethanolamide (PEA), and oleoylethanolamide (OEA) have been reported in ASD children compared to neurotypical controls.

The therapeutic potential of ECS modulation is under investigation. Cannabidiol (CBD), a non-psychotropic cannabinoid, has been shown to be promising in alleviating core ASD symptoms and related comorbidities [110]. Since ECS signaling is closely integrated with other neurotransmitter systems (i.e., serotonergic, GABAergic), which are also dysregulated in ASD, this endogenous machine could be a key target for therapeutic intervention. Beyond the central nervous system (CNS), the ECS is also a critical component of the MGBA. The peripheral gut ECS communicates with the central brain ECS, involving peripheral nerves, the enteric and CNS, gut hormones, the intestinal microbiota, and microbiota-derived molecules [111].

Studies in germ-free (GF) mice have shown that the gut microbiome modulates ECS tone, impacting intestinal permeability and systemic inflammation. For instance, microbiota depletion or alteration reduces intestinal levels of ECS mediators and alters the expression of related genes, with functional consequences on neurodevelopment and behavior. Notably, fecal microbiota transfer (FMT) from conventionally raised mice to GF mice restores ECS signaling in the gut, particularly in the ileum and jejunum [112,113]. Stress-related changes in ECS signaling further link microbiota, emotion, and cognition. In models of chronic stress, decreased AEA and altered 2-AG levels have been associated with activation of the hypothalamic–pituitary–adrenal (HPA) axis, anxiety-like behavior, and impaired synaptic plasticity.

Transferring microbiota from stressed animals to naïve mice replicates these effects and induces ECS downregulation, which can be reversed by dietary supplementation with 2-AG precursors or MAGL inhibition [114]. Taken together, these findings highlight the ECS as a central node in the pathophysiology of ASD, bridging genetic, neurochemical, and environmental influences. Its interactions with the gut microbiota and stress systems further underscore its potential as a therapeutic target in autism and related neurodevelopmental disorders.

### 4.2. Intestinal Permeability and ASD

The intestinal epithelial barrier helps maintain biological homeostasis by separating the internal and external environments. It limits the infiltration of exogenous agents and the leakage of endogenous substances [115]. Intestinal permeability refers to the property that allows the exchange of solutes and fluids between the intestinal lumen and the mucosa [116]. “Leaky gut” describes a condition in which the intestinal epithelial barrier is compromised, and intestinal permeability is increased, allowing molecules and cells to pass between the gut and the circulatory system. The concept of “leaky gut” has been recognized as an important factor in both intestinal and extra-intestinal disorders [117].

Children with ASD frequently experience GI problems and, since GI symptoms occur in up to 70% of ASD cases, the MGBA is likely to play a significant role in ASD [1]. Recent studies have shown that a significant proportion of individuals with ASD have higher intestinal permeability than the general population [118,119]. For example, 36.7% of children with ASD and GI symptoms were reported to have impaired intestinal permeability [120].

The severity of ASD also appears to be correlated with GI symptom prevalence, suggesting a role of gut dysfunction in ASD etiology [121], as described in the following paragraphs. Leaky gut syndrome has been proposed as a marker of enteropathy in ASD children and has been confirmed over the past decade [122]. Increased intestinal permeability has been reported in 43% to 76% of children with ASD, with or without GI symptoms [123]. Moreover, Teskey et al. (2021) found that the intestinal fatty acid-binding protein (I-FABP), a marker of epithelial damage, is associated with more severe behavioral symptoms in ASD children [118].

A 2022 study by Piras and collaborators showed that increased intestinal permeability in ASD children, indicated by urinary metabolites, such as fucose, phenylacetylglycine, nicotinurate, and 1-methylnicotinamide, is linked to gut dysbiosis, selective eating, and mucosal inflammation. These factors, along with physical barrier disruption, facilitate the entry of microbial metabolites and toxins into the systemic circulation, negatively affecting the MGBA and contributing to ASD symptoms [124].

In mouse models of ASD, such as BTBR T + Itpr3tf/J and VPA, Simeng Liu and research partners have recently observed impaired intestinal barrier function, including increased permeability, the downregulation of tight junction proteins (claudin-1, claudin-3, and occludin), and elevated pro-inflammatory cytokines (IL-6, TNF-α, IFN-γ). Notably, the probiotic, *Clostridium butyricum*, was shown to enhance intestinal barrier integrity, reduce the expression of histone deacetylase 1 (HDAC1), and improve ASD-related behaviors in BTBR mice. This effect appears to involve the Trek1 potassium channel, suggesting that it could be a downstream target of butyric acid signaling. These findings support the therapeutic potential of microbiota-targeted interventions in ASD [125].

Beyond preclinical evidence, clinical studies also support barrier dysfunction in ASD [126]. Altered intestinal and blood–brain barrier (BBB) permeability has been implicated in the pathophysiology of ASD. Key structural proteins involved in maintaining these barriers include claudin-5, claudin-11, occludin, β-catenin, vinculin, and paxillin. A recent study assessing serum levels of these proteins in preschool-aged children with ASD revealed significantly elevated concentrations of claudin-11, occludin, and β-catenin compared to typically developing controls, while claudin-5, vinculin, and paxillin showed no significant differences. These results suggest a potential role for specific barrier-associated proteins in ASD, possibly through the modulation of intestinal or BBB permeability or alternative neuroimmune mechanisms [126].

Higher serum zonulin levels, another physiological regulator of intestinal permeability, were detected in 32 patients with ASD compared to the healthy controls [127]. Moreover, zonulin content in intestinal and serum samples seems to be positively correlated with symptom severity, suggesting a potential role of intestinal barrier dysfunction in the pathogenesis of ASD [128].

### 4.3. Inflammatory Markers in ASD

Among the mechanisms that may be involved in ASD, there has been increasing interest in the dysregulation of the immune system [129,130].

Cytokines are a group of small proteins that play essential roles in regulating inflammation and neurodevelopment as messengers that modulate the immune system and endocrine glands [131,132].

In the CNS, cytokines are produced by neurons and glia, or they reach the CNS from the periphery through several pathways. Cytokines can pass via the BBB’s leaky regions; the brain can recognize pro-inflammatory cytokines, such as IL-1α, IL-1β, TNF-α, and IL-6 as molecular signals of disease. In addition, the cytokines TNF-α, IL-6, and IL-1β cross the BBB and act on the hypothalamus, promoting fever and sickness behaviors [133,134]. Cytokines can be actively transported via specific transport molecules; they can also be transmitted via cranial nerves and released by transferred peripherally activated monocytes [133].

To date, a wide range of studies have highlighted that cytokines can be considered as biomarkers in ASD.

Emerging evidence highlights immune dysregulation as a key component in the pathophysiology of ASD, with numerous studies reporting altered levels of inflammatory markers. Among these, the C-reactive protein (CRP) is an acute phase protein that reflects systemic low-grade inflammation. Elevated CRP levels in ASD have been associated with increased concentrations of pro-inflammatory cytokines, such as IL-6, suggesting an active inflammatory state [134]. IL-6 plays a critical role in neurodevelopment and its overexpression has been linked to disruption in the excitatory and inhibitory synaptic balance and aberrant dendritic spine morphology, which may underlie ASD-related behavioral phenotypes [134].

The importance of cytokine profiles in ASD has been recently reported, showing that immune system abnormalities, with elevated levels of IL-6 and TNF-α, in children with ASD may impair neuronal signaling and development [135]. However, anti-inflammatory cytokines, such as IL-10, appear to be reduced in ASD. IL-10 is a key immunoregulatory molecule that limits inflammation and prevents tissue damage; its downregulation in ASD patients suggests impaired immune homeostasis [134,135]. Similarly, IL-8 has emerged as a particularly promising biomarker. In a family triad-based case-control study, Shen et al. showed that the levels of this pro-inflammatory chemokine involved in immune cell recruitment were significantly associated with ASD severity, correlating with specific social domains, such as awareness, cognition, and motivation [131], pointing to dysregulation in both pro- and anti-inflammatory signaling pathways [131,134].

Ferencova et al. investigated cytokine levels in adolescents with ASD [132]. Their study revealed elevated levels of both pro-inflammatory (IL-1α, IL-1β, IL-2, IL-6, IL-8) and anti-inflammatory (IL-4, IL-10) markers, alongside increased white blood cells and monocytes. This dual activation of inflammatory and compensatory immune pathways suggests a complex, possibly sex-dependent, immunological profile in ASD adolescents of both genders [132]. The correlation between CRP and both IL-10 and IL-8, observed by Noori et al., further supports the hypothesis of immune activation in ASD [134]. This interplay between systemic and local inflammatory responses may reflect underlying neuroimmune alterations.

Beyond cytokines, recent research has explored broader inflammatory pathways. A pilot interventional study assessing the effects of a modified ketogenic diet (KD) in children with ASD reported decreased plasma levels of IL-1β and IL-12p70 after the dietary intervention, along with reduced BDNF and modulation of gut microbiota [136]. These findings suggest that diet-induced changes in inflammation and microbiota composition may influence ASD symptomatology.

Proteomic studies have added a further level of detail to these findings. Using Olink proteomics, Bao et al. identified thirteen differentially expressed inflammation-related proteins in ASD children compared to the controls [137]. Among these, STAMPB, ST1A1, SIRT2, and MMP-10 demonstrated good diagnostic performance. Combinatory biomarker models showed even higher accuracy and correlations with clinical features, such as age and parity, which suggest that inflammation in ASD may be influenced by developmental and environmental factors [137].

Taken together, these findings support the role of immune dysregulation in ASD, involving both peripheral and central inflammatory pathways. Altered levels of cytokines, like IL-6, IL-8, and IL-10, along with systemic markers, such as CRP and proteomic signatures, underline the relevance of inflammation in the disorder’s etiology and progression.

Inflammatory biomarkers offer valuable insights into the immunopathological landscape of ASD and may serve as viable candidates for early diagnostic application and individualized therapeutic interventions. Thus, these data reinforce the emerging view that immune dysregulation constitutes a core component of ASD pathophysiology.

### 4.4. Intestinal Microbiota

The intestinal microbiota is a large community of microorganisms that inhabit the human GI tract, with a prevalence of bacteria, but also fungi, viruses, and protozoa [138]. The composition of this microbial ecosystem is influenced by genetic factors, diet, lifestyle and environment, and is subjected to continuous variations [139]. In addition to playing a key role in digestion, nutrient metabolism, and vitamin synthesis, the gut microbiota has profound effects on multiple physiological systems, particularly on the gut–brain axis, a bidirectional communication system that connects the central nervous system (CNS) with the enteric nervous system through neural, immune, endocrine, and metabolic pathways [111].

In recent years, attention towards the intestinal microbiota and its impact on ASD has notably grown. Although the exact etiopathogenesis of ASD is poorly understood, in recent decades research has highlighted the interaction between the intestinal microbiota and the brain in patients with autism and/or other neuropsychiatric diseases. The intricate relationship between the gastrointestinal (GI) tract, the microenvironment within the gut, and the CNS is referred to as the MGBA [140], also known as “our second brain” [141].

In that regard, a growing body of experimental evidence has demonstrated a link between changes in the composition of the gut microbiota and ASD symptoms, highlighting how alterations in the intestinal microbiota can influence both GI and neurobehavioral symptoms in patients with ASD [142]. An imbalance in the intestinal microbial composition, known as dysbiosis, in ASD has been widely demonstrated [143,144]; however, a characteristic profile of the microbiota composition in subjects with ASD has not been identified yet. Specific taxa, *Bacteroides, Clostridiaceae*, *Enterobacteriaceae,* and *Lactobacillales*, have been found to be enriched in ASD mice [11]. Changes in the gut microbiota have also been observed in individuals with ASD [145].

Recent studies have shown that some bacteria belonging to the phylum *Bacteroidetes* [*Barnesiella*, *Parabacteroides*, *Bacteroides*, *Odoribacter*, *Prevotella*, *Proteobacteria* (i.e., *Proteus* and *Parasutterella*) and *Alistipes*] are more abundant in ASD patients compared to the general population, while Actinobacteria, (*Bifidobacterium* spp.) are often find decreased in ASD [41]. GI symptoms in ASD children, such as constipation, have been associated with the abundance of *Escherichia/Shigella*, *Clostridium* cluster XVIII [142], and other genera, such as *Fusobacterium*, *Barnesiella*, *Coprobacter*, *Olsenella*, and *Allisonella* [146], and a lower abundance of beneficial microbes, such as *Faecalibacterium prausnitzii* and Lactobacilli [145,147].

Dysbiosis related to the ratio of *Firmicutes* to *Bacteroides*, as well as the number of species of the *Firmicutes*, *Bacteroidetes*, *Fusobacteria*, and *Verrucomicrobia* phylum, has been demonstrated in patients diagnosed with ASD [148]. Other studies have confirmed that ASD patients present a lower relative abundance of various bacteria, including the genera *Bifidobacterium, Prevotella, Enterococcus, Ruminococcus,* and *Coprococcus* [149], and a reduced ratio between the phylum *Bacteroidetes* and *Firmicutes* [142], commonly used as an index of eubiosis/dysbiosis [150].

Previous studies have highlighted how an imbalance between these two phyla can also be associated with several other pathological conditions, including obesity, inflammatory bowel diseases, and metabolic disorders [151]. Studies in animal models have shown that dysbiosis can alter synaptic plasticity and neurogenesis, negatively affecting mood and cognitive function [152].

Moreover, the microbiota produces a wide range of bioactive metabolites, such as short-chain fatty acids (SCFAs), especially acetate, propionate, and butyrate, derived from the fermentation of dietary fiber. These SCFAs can cross the BBB and directly influence the CNS, modulating inflammatory processes and neurotransmitters levels, as serotonin and dopamine involved in early brain development [153]. It has been reported that, in ASD patients, changes in the intestinal microbiota composition led to alterations in SCFA levels, which were restored by the administration of butyrate, that, in turn, improved ASD symptoms [154]. Furthermore, reduced levels of SCFAs can have an impact on both intestinal and BBB integrity, promoting neuroinflammation, with the risk of direct impairments to brain functions [155].

In addition, the microbiota is also capable of directly synthesizing neurotransmitters, such as serotonin, dopamine, GABA, and other molecules, that through the vagus nerve, directly communicate with the brain, regulating mood and cognitive function [156,157]. Moreover, changes in the structure and composition of intestinal microbiota directly influence the levels of volatile organic compounds (VOCs), such as indole, a metabolite of tryptophan and a precursor of serotonin and melatonin [158]. Indeed, it is well-known that intestinal bacterial populations also play a crucial role in the regulation of neurotransmitter precursors, especially by increasing tryptophan levels in plasma [159] and/or by producing SCFAs, that, in turn, leads to the stimulation of the serotonergic system [160].

Intestinal dysbiosis may also contribute to the systemic inflammatory state reported in ASD patients with GI comorbidities, by increasing intestinal permeability, leading to a leaky gut. In ASD, such increased permeability may be linked to changes in microbiota composition, including a reduction in *Lactobacillus* spp., which are known to support epithelial barrier integrity [121]. Indeed, this condition is characterized by an imbalance of gut promoter taxa (i.e., Lactobacilli and Bifidobacteria) that may facilitate the transit of pro-inflammatory molecules and toxins into the bloodstream, which, in turn, could reach the brain, worsening neurological symptoms [122]. It has been shown that gut dysbiosis can promote a chronic low-grade inflammatory state, contributing to conditions such as major depression, which has been associated with increased levels of pro-inflammatory cytokines [161]. This highlights the importance of the microbiota not only in maintaining the gut balance, but also in regulating inflammation at the systemic and brain levels, suggesting the manipulation of the gut microbiota as a key treatment strategy in ASD [162].

However, it is important to consider that these data could be influenced by the antibiotic treatment and/or personalized diet of ASD patients [14]. Chronic inflammation of the intestinal tract, found in many patients with ASD, is also correlated with adverse reactions to specific foods components, such as gluten and casein, which have led many families to adopted exclusion diets [163].

On the other hand, due to the strict interplay between foods, intestinal microbiota, and human health, diet can rebalance the gut microbiota composition, can directly modulate the gut–brain axis, and can positively affect brain functioning, representing a promising key strategy in ASD intervention [164]. As, for example, emerging research is focusing on the potential impact of plant food bioactive molecules and their metabolites (i.e., polyphenols, dietary fibers, omega-3-fatty acids etc.), which are already well-known for their recognized antioxidant, anti-inflammatory, and immunomodulatory properties, on neuroprotection in ASD [164]. From this perspective, plant-based fermented foods represent a huge source of bioactive and neuroprotective molecules [165,166]. Although some studies have reported symptomatic improvements as a result of these diets, their effectiveness has not yet been conclusively confirmed through large randomized clinical trials [167].

Gut microbiota disorders in individuals with ASD have also attracted increasing scientific interest for their possible role in exacerbating social and behavioral symptoms [168]. As already mentioned, children with ASD suffer from GI symptoms, such as constipation, diarrhea, abdominal pain, and gastroesophageal reflux [1]. These symptoms not only influence their quality of life, but also appear to be correlated with typical autism behaviors, such as irritability, anxiety, and repetitive behaviors [140,169]. Scientific research on the MGBA suggests that this microbial ecosystem plays a fundamental role in mental and neurological health, as well as influences many aspects of human physiology and behavior [111,162].

Further research is needed to clearly define the role of the gut microbiota in ASD patients and to fully understand its relationships with ASD. Maintaining or restoring a healthy intestinal microbiota is, therefore, of utmost importance to prevent the pathology from manifesting itself or to soothe the symptoms of the pathology.

## 5. Probiotics in ASD

Probiotics, generally belonging to Gram-positive microbial groups, such as Lactobacilli and Bifidobacteria, are defined as “live, viable microorganisms that confer a health benefit to the host when administered in adequate amounts” [170].

So far, probiotics have been largely administered for a wide range of demonstrated health benefits, including the maintenance of gut homeostasis, mainly by modulating gut microbiota, preventing and/or restoring gut dysbiosis, interacting with intestinal epithelium, and stimulating the enteric and systemic immune system [171]. In the last decade, an interesting class of probiotics, so-called psychobiotics, has emerged due to their potential to specifically influence mental health by acting on the gut–brain axis. This term was first coined by Dinan et al. (2013) to describe those strains of bacteria that are able to modulate the intestinal microbiota, with positive effects on psychiatric disorders, such as anxiety and depression [172].

Recent studies have shown that children with autism tend to have a reduced abundance of beneficial bacteria, such as *Bifidobacterium* spp. and *Lactobacillus* spp., and an increase in potentially pathogenic bacterial species [173]. This imbalance may contribute not only to the GI disorders common in ASD, but may also influence behavioral and neurological symptoms, indicating microbiota manipulation in ASD using probiotic administration as a potential therapeutic strategy to improve both behavioral and GI symptoms [174,175].

Moreover, probiotics have been suggested as potential reducers of the inflammatory state, by reducing the state of intestinal hyperpermeability, induced by intestinal dysbiosis associated with ASD [176,177]. A clinical study involving children with ASD demonstrated that probiotic supplementation can significantly improve levels of zonulin, a protein regulating intestinal permeability, which is considered to be a key biomarker of intestinal barrier, along with occludin and fecal calprotectin [178,179].

Furthermore, the main psychobiotics, including Lactobacilli and Bifidobacteria and their metabolites (recently defined as metabiotics) [180,181], can directly influence the CNS via the gut–brain axis, through the production and/or stimulation of different types of neurotransmitters (i.e., serotonin, GABA, acetylcholine, dopamine, and noradrenaline) and by modulating the metabolism of their precursors, such as tryptophan [159,182], often found to be altered in ASD patients [164,180].

Moreover, the combination of prebiotics/probiotics is emerging as a promising alternative strategy. As, for example, a dietary intervention with probiotics and fructo-oligosaccharide (FOS) significantly restored SCFA and serotonin levels, improving neurotransmitter disorders, as a hyper-serotonergic state, and altered dopamine metabolism, that, in turn, ameliorated symptoms in ASD children [183].

Recently, bacteria isolated from different foods also showed similar capabilities in regard to modulating neuroactive molecules. Among them, some Lactobacilli, mainly belonging to *Lactiplantibacillus (Lpb.) plantarum* species, have been reported for their health benefits, improving stress, anxiety, and cognitive functions through the stimulation of dopamine and serotonin pathways [184,185,186].

A recent review by Diamanti et al., 2022, reported preclinical and clinical evidence on the use of probiotics from human and food origins in restoring neurotransmitter levels in diverse neurodevelopmental diseases, including ASD [14].

The efficacy of probiotics in improving ASD symptoms has been the subject of several clinical studies [175] and a recent updated systematic review and meta-analysis involving more than 300 samples from young ASD patients (1.5–20 y.o.), which clearly suggests that probiotic administration is a successful dietary intervention to significantly improve behavioral symptoms [187].

Despite these promising results, probiotic therapy for ASD is not without limitations. One of the main obstacles is the individual variability in response to probiotics, which may depend on factors such as pre-existing gut microbiota composition, genetics, diet, and lifestyle [188]. Furthermore, not all probiotics are the same. The selection of the most effective strains, along with the dosage, mode, and time of administration, are crucial aspects to obtain consistent therapeutic results [146].

### Lpb. plantarum and Health Benefits in ASD

Among all the probiotics within the Lactobacilli group, *Lpb. plantarum* has attracted great interest for its potential functional activities and is one of the most studied probiotic species preventing and/or ameliorating a broad spectrum of diseases from chronic intestinal inflammation (i.e., IBD), infections, and cancer to maternal and neonatal disorders [189,190]. More recently, *Lpb. plantarum* has also emerged as a promising psychobiotic, with positive effects on neurodevelopmental and neurodegenerative conditions through the modulation of gut microbiota, its interaction with the gut–brain axis, and the regulation of intestinal and blood–barrier integrity [191].

*Lpb. plantarum* is a very flexible and versatile species that is found in several fermenting foods, such as dairy products, cured meats, different types of vegetables, wine, and baked goods [192]. Furthermore, it is a natural inhabitant of the oral cavity, intestine, and genital tract of humans and other animals [193].

In addition, the food origin of *Lpb. plantarum* confers easy availability and safety for daily use, facilitating its integration into the diet through fermented foods or probiotic supplements. Its ability to adapt to variable environmental conditions, such as low stomach pH and the presence of bile salts in the intestine, makes it a valid probiotic candidate, able to effectively colonize the intestine and exert its beneficial activities [194]. Indeed, food-borne strains of *Lpb. plantarum* may also play a significant role in improving intestinal and mental health [193]. In addition to neuropsychiatric effects, Garcia et al. highlighted the importance of food-derived *Lpb. plantarum* strains in supporting neurological and behavioral conditions [193].

These strains with probiotic potential, already part of many dietary traditions, can therefore offer a natural and functional approach to improve not only intestinal health, but also mental and neurological health [191].

Table 2 provides information on the available preclinical studies involving diverse ASD animal models that have shown the potential positive impact of different strains of *Lpb. plantarum,* alone and/or in combination with other probiotics, in ameliorating ASD symptoms in animal models by acting on intestinal homeostasis and in improving autism-like behaviors.

In particular, *Lpb. plantarum* ST-III improved the impaired social interaction and autistic-like behaviors in two mouse models of ASD by modulating specific gut microbes [169,201]. In the study conducted by Guo et al. [169], *Lpb. plantarum* ST-III was able to modulate the gut microbiota of the offspring of pregnant mice exposed to triclosan by specifically increasing *Lachnospiraceae*, recognized to be a family of beneficial microbes for human health, mainly as SCFA producers [202], and simultaneously reducing the abundance of *Alistipes*, which are involved in the conversion of tryptophan into indoles, leading to a serotonergic imbalance [203]. Changes in the gut microbiota can regulate abnormal behavior in mouse models of ASD, by restoring altered amino acid biosynthesis, by producing the precursors of metabolites and energy and helping the biosynthesis of nucleosides and nucleotides [169].

Similar results have been shown after the administration of the probiotic, *Lpb. plantarum N*-1, in a MIA mouse model of ASD [196]. Another study in animal models showed that probiotics supplementation can modulate the immune system by reducing pro-inflammatory cytokine levels (IL-6 and IL-17A), thus improving the neuroinflammatory balance associated with ASD in adult MIA offspring [201].

Promising results have been obtained after the combined intervention of *Lpb. plantarum* and prebiotics, such as inulin, arabinogalactan, and β-glucan (BTAGEN^®^), in decreasing intestinal inflammation and improving immune functions in VPA Wistar rats [198,199]. Prebiotics, such as inulin, selectively promote the growth and increase in the abundance of Lactobacilli and Bifidobacteria [204], offering synergistic health-promoting effects when combined with probiotics [205]. The use of combined probiotic/prebiotic interventions in alleviating ASD symptoms has also been confirmed in clinical studies, showing multiple effects such as modulation of the gut microbiota, increasing SCFAs and serotonin, along with an improvement in hyper-serotonergic states and dopamine metabolism [183]. These results have confirmed precise dietary interventions as a promising strategy in ASD treatment.

Moreover, the *Lpb. plantarum* PS128 strain has been shown to improve sociability, anxiety, and cognition in VPA mice by improving synaptic plasticity, the dendritic structure, and glutamate receptor expression [197]. The anxiolytic and antidepressant properties of this strain are due to its ability to affect the production of key neurotransmitters, such as serotonin, dopamine, and GABA, all of which are crucial for regulating mood and emotions [206]. Through this mechanism, PS128 can reduce symptoms related to anxiety and depression, contributing to improving mental well-being.

In particular, the PS128 strain has shown promising results in clinical models of ASD and ADHD, improving social functions and reducing impulsive behaviors [146,207].

This effect could be mediated by the ability of *Lpb. plantarum* to reduce systemic inflammation and regulate the immune response, processes that play a crucial role in neuroinflammatory diseases and neurodevelopmental disorders. In addition, the strain has shown a positive impact in modulating the HPA axis, contributing to the reduction of chronic stress [207].

The application of *Lpb. plantarum* as an alternative therapy against ASD has also recently been investigated in clinical trials (Table 3). As reported in Table 3, recent studies have shown that the intestinal microbiota of children with ASD is often characterized by an increase in pro-inflammatory bacteria and a decrease in beneficial species, thus, Lactobacilli supplementation, such as *Lpb. plantarum*, could restore this balance, improving both GI and behavioral symptoms.

Up to date, the available four randomized and double-blind clinical trials [146,208,209,210], investigating the impact of oral *Lpb. plantarum* PS128 administration (3–6 × 10^10^ CFU/day) in ASD children, showed that *Lpb. plantarum* PS128 supplementation for around 4 months improved GI symptoms, reducing constipation and bloating in children with ASD and, in line with the published literature [197], led to behavioral improvements.

Furthermore, *Lpb. plantarum* appears to have neuroprotective effects through the production of GABA, a neurotransmitter that regulates mood and anxiety, both of which are often altered in patients with ASD [206]. The association between the intake of *Lpb. plantarum* PS128 and behavioral improvements, particularly in the case of comorbid GI symptoms, was also confirmed in adolescents in a recent retrospective observational study, enrolling 131 autistic patients [207].

Interestingly, promising results have also been obtained in clinical trials using a combination of eight different probiotic strains (so-called Vivomixx^®^ in the EU, Visbiome^®^ in the USA), which also includes *Lpb. plantarum* [179,211,212,213]. In particular, the administration of this probiotic formulation improved social affect scores in children without GI issues, and enhanced GI sensory and adaptive functions in those with GI symptoms [213], and restored typical brain activity, suggesting neuroplastic effects and gut–brain modulation in preschool children with ASD [179,211]. Similar results have also been obtained by the administration for 4 weeks of a diverse multi-strain combination of 10 probiotics (#VSL3), showing the amelioration of GI symptoms and ASD core symptoms in a 12-year-old child with ASD [214]. These results are in line with the increasing tendency to prefer probiotics mixtures made up of a high number of different strains, in order to enhance efficacy, due to the potential synergistic effects [215].

The question of whether multi-strain probiotics confer superior efficacy compared to single-strain formulations is still open for debate, with conflicting findings reported across studies. Consequently, evidence from clinical trials continues to serve as the primary criterion for guiding the selection of appropriate probiotic interventions [215,216]. As, for example, Tomova et al. [178] emphasize that the efficacy of probiotic *Lpb. plantarum* may also vary depending on the patient’s microbiota composition and individual characteristics. Indeed, a recent analysis on the efficacy of using probiotics, including *Lpb. plantarum,* in ASD children, reported no consistent outcomes, in randomized clinical studies [217].

More recently, the systematic review and meta-analysis of seven probiotic intervention studies conducted by He et al., 2023, showed major limitations in regard to the small sample size and short-term administration, along with the use of different probiotics strains and poor research quality [218]. Therefore, there is a need to increase the number of well-designed and large-scale, randomized, double-blind, and placebo-controlled clinical trials to better understand the mechanisms of action, optimal dosages, and duration of treatment, in terms of the efficacy of single *Lpb. plantarum* and multi-strain formulation, as a part of an integrated therapeutic approach to ASD.

## 6. Conclusions

Understanding ASD remains a major challenge for the scientific community, as its multifactorial nature requires multidisciplinary research and therapeutic approaches. In this context, the study of a bunch of different biomarkers, such as those related to the ECS, intestinal permeability, inflammation, and gut microbiota, has provided crucial information in order to understand the complex pathophysiology of ASD.

These findings highlight the therapeutic potential of targeting gut barrier integrity and microbiota composition to alleviate both GI and behavioral symptoms in ASD, paving the way for microbiota-based interventions and biomarker-driven approaches in future treatment strategies.

Although among novel therapeutic approaches, probiotic supplementation, particularly the use of Lpb. plantarum strains, is promising, further large-scale and additional clinical trials are needed to define optimal strain formulations, dosages, and treatment regimens, suitable for clinical standardization. Importantly, future strategies should also account for individual variability, including sex, gender, and age at diagnosis, to enable the development of truly personalized interventions for ASD.

## Figures and Tables

**Table 1:** Summary of the applied genetic, environmental, and idiopathic in vivo models of ASD.

Symbol	Name	Model Category ^1^	Number of Models	Behavioral Features	Neuroanatomical Alterations	Gastrointestinal Alterations	References
SHANK3	SH3 and multiple ankyrin repeat domains 3	G	111	↓ Social interaction and communication; repetitive behaviors.	↓ Synaptic transmission; altered functional and structural plasticity of synapses; ↑ dendritic length and complexity; ↓ spine density.	↓ Intestine relative abundance of members of the class *Bacilli*, order *Lactobacillales*, family *Lactobacillaceae*, and genus *Lactobacillus.*	[54,55].
MECP2	Methyl-CpG binding protein 2	G	95	Motor impairment such as ataxia; anxiety-like and anti-social behaviors; stereotyped behaviors.	↓ Volume of cortical and subcortical regions; altered synaptic transmission.	Short colon; abnormal localization of key membrane proteins, like ClC-2 and NHE-3, in the epithelial cells on the surface.	[28,56,57].
FMR1	Fragile X mental retardation 1	G	68	↑ Locomotor activity; hyperactivity; ↑ self-grooming; ↑ repetitive behaviors; ↓ anxiety (due to the background).	↑ Spine density and length; abnormal synaptic plasticity; ↓ ratio of AMPA to NMDA receptors early in development.	Altered gut microbiota composition; ↑ intestinal inflammation; ↑ intestinal permeability; ↑ serum LPS levels.	[58,59].
CNTNAP2	Contactin associated protein-like 2	G	52	Social deficits; communication impairment; repetitive behaviors.	Defective neuronal migration and cortical ectopia; altered sensory cortical circuitry; long-range connectivity deficits; dendritic spine morphology and synaptic plasticity deficits.	Altered colonic motility; ↑ intestinal permeability.	[60,61,62,63,64].
NLGN3	Neuroligin 3	G	50	Abnormal social and repetitive behaviors.	Altered excitatory synaptic transmission; alterations in synaptic signaling and plasticity; glial cell morphology changes.	Accelerated gastrointestinal transit and dysmotility; alterations in enteric nervous system (ENS); altered mucus layer and microbiota distribution.	[65,66,67,68,69].
CHD8	Chromodomain helicase DNA-binding protein 8	G	48	Impaired social interaction; anxiety; learning and memory deficits.	↑ Brain weight, craniofacial abnormalities; altered synaptic physiology in medium spiny neurons of the nucleus accumbens; microstructural changes in specific brain regions, including the cortex and striatum.	Shortened small intestine and colon length; ↓ intestinal motility; disturbance in the gut microbiota, including decreased abundance of Bacteroides.	[29,30,70].
PTEN	Phosphatase and tensin homolog	G	45	↓ Social preference; ↓ social novelty; ↓ aggression; repetitive behaviors.	Macrocephaly; ↑ glia (astrocytes, oligodendrocytes, and microglia) neuronal hypertrophy; ↑ axon growth.	//	[31].
TSC1	Tuberous sclerosis 1	G	40	Impairments in social interaction and communication; restricted and repetitive behaviors.	Cortical and hippocampal hypertrophy; brain structural abnormalities; abnormal synaptogenesis; glial cell overexpression; neuroinflammation; oxidative stress; mitochondrial dysfunction (cerebellum, cortex, hippocampus, amygdala).	//	[32,71,72].
UBE3A	Ubiquitin protein ligase E3A	G	34	Impairments in social interaction; repetitive self-grooming behavior; memory and pain sensitivity.	↓ Dendritic spine density and ↑ immature filopodia density, alterations in neurons as immature dendritic protrusions; reduction in dendritic spine maturation in prelimbic cortical neurons.	//	[33,73,74].
TBR1	T-box, brain 1	G	31	↓ Social behaviors; defective vocalization; impaired olfactory discrimination; aversive memory; and cognitive inflexibility.	Impairments in structural and functional connectivity of the basolateral amygdala, and whole-brain synchronization.	//	[34,75].
Poly I:C	Polyinosinic–polycytidylic acid	E	71	Sensorimotor gating; perseverative behaviors; ↓ social interaction, abnormal communication, stereotyped/repetitive behavior.	Spatially restricted deficit in Purkinje cells, neuroinflammation, alterations in BDNF and ARG-1 levels.	Altered early-life gut microbiota composition, gut dysbiosis, ↑ intestinal permeability, ↑ bacterial families such as *Lachnospiraceae*, *Porphyromonadaceae*, and *Prevotellaceae.*	[37,45,46,76,77]
VPA	Valproic acid	E	36	↓ Sociability, communication deficits, repetitive behaviors; impaired prepulse inhibition; altered pain sensitivity, ↑ anxiety, and hyperactivity.	Differences in head circumference or brain size; macrocephaly and microcephaly; ↓ myelin density or gene/protein expression.	Impaired duodenal motility; ↑ intestinal inflammatory factors.	[36,78,79,80,81].
GF environment	Germ-free environment	E	7	Repetitive behavior; ↓ sociability; ↓ anxiety; non-spatial memory.	↑ Neuroendocrine responses to stress; altered neurotrophin levels in the hippocampus and amygdala; altered monoamine neurotransmitter levels in the brain.	↓ Total mass of intestine; larger cecum; reduced numbers of gut-associated lymphoid tissues, poorly formed T-cell and B-cell zones in the germinal centers; reduced numbers of intestinal T cells and decreased IgA production.	[82,83,84]
MHFD	Maternal high-fat diet	E	6	Impaired sociability; prepulse inhibition learning and memory impairment; hyperactivity; enhanced anxiety-like behaviors; ↓ cognition.	↓ Oxytocin immunoreactive neurons in the hypothalamus; block long-lasting neural adaptation in the mesolimbic dopamine reward system; attenuations of amino acid levels in the medial prefrontal cortex and the hippocampus regions.	Changes in microbiome composition, ↓ *L. reuteri*; disruption of intestinal mucosal barrier.	[39,40,83,85]
rIL-6	Recombinant interleukin 6	E	6	Impaired sociability; repetitive behaviors; cognitive and learning abnormalities; prepulse inhibition; latent inhibition.	↑ Brain volume; alterations in dendritic spine; imbalance between excitatory and inhibitory synapses.	↑ Intestinal permeability; alterations in microbiota composition.	[86].
Fluoxetine	Fluoxetine	E	4	Anxiety behaviors; disrupted learning; aggressive behaviors; impaired social recognition and working memory.	Abnormal circuit formation in the cortex; ↓ frequencies in spontaneous excitatory postsynaptic currents recorded from layer (L) 5 pyramidal neurons in the prelimbic cortex.	Impaired enteric neuronal development; altered GI motility and mucosal growth; hyperplastic enteric system; ↑ intestinal transit; ↓ *Lactobacillus johnsonii* and *Bacteroidales S24-7*.	[48,87,88,89]
Stress	Stress	E	4	Social interaction impairments; conditioned fear behavior; alterations in anxiety-like behavior.	↑ Brain 5-HT; precocious synaptic maturation (hippocampus); ↓ neuronal arbor complexity and synaptogenesis; ↑ CLDN5, CLDN12, MMP9 (PFC); ↓ CA1/CA3 hippocampal diameter.	↓ In alpha and beta diversity of microbiota; ↓ in fecal propionic acid; intestinal inflammation; gut microbiota dysbiosis; ↓ ocln and cldn1 expression in the colon.	[49,90,91,92]
LPS	Lipopolysaccharide	E	3	Deficits in social interaction; novel object recognition; anxiety-like behavior.	Structural, neurophysiological, and functional changes in the hippocampus; abnormal fetal brain cytoarchitecture and lamination; activation of microglia in the fetal brain.	Intestinal inflammation.	[93,94,95]
Prenatal stress	Prenatal stress	E	3	↓ Sociability, ↓ reciprocal social interaction; ↑marble burying behaviors; ↑anxiety; rigid response learning strategy; impaired memory in a motor learning task.	Aberrant expression of glutamate and GABA marker genes; disorganization of striatal striosome and matrix compartments; neuroinflammation; ↓ oxytocin receptor; ↓ serotonin metabolism in the cortex.	Gut dysbiosis; ↓ *Bacteroides* and *Parabacteroides*; impaired intestinal epithelial proliferation, goblet and Paneth cell differentiation; mucosal and gut barrier dysfunction; low-grade inflammation; ↓ ileum villus height, crypt depth, and surface area.	[50,81,96,97,98,99]
BALBcByJ	BALB/cByJ	I	24	Social behavior impairment; ↑ repetitive and stereotypic activities; ↓ ultrasonic vocalizations; heightened anxiety-like behaviors; excessive grooming.	↓ Corpus callosum volume; ↓ fractional anisotropy (FA) in the external capsule area, indicative of decreased integrity of white matter fibers.	//	[53,100].
BTBR	BTBR T + Itpr3tf/J	I	73	Impaired sociability; repetitive behaviors; abnormalities in vocal communication.	↓ Gray matter volume in ventral tegmental area, cingulate gyrus, lateral thalamus, posterior thalamus, occipital and parietal cortices, and subcortex; ↑ gray matter volume in olfactory bulb, medial prefrontal and insular cortices, amygdala, and dorsal hippocampus.	↑ Intestinal permeability; downregulation of Muc 2 in the large intestine; altered microbiota composition of cecal and fecal samples; ↓ *Bifidobacterium* and *Blautia* species.	[51,52,95,100,101,102].

^1^ The model categories are synthetized as genetic (G), environmental (E), and idiopathic (I).

**Table 2:** Preclinical studies involving probiotic *Lpb. plantarum* administration in ASD animal models.

Animal Model	Treatment	Age/Sex	Behavioral and Physiological Features	Main Outcomes	Relevance to Human ASD	Reference
ICR mouse (TCS exposure).	*Lpb. plantarum* ST-III.	6–9 weeks/ M and F.	Social deficits (males); grooming and freezing (females); altered gut microbiota composition.	*Lpb. plantarum* ST-III improved social behavior in males, reduced grooming/freezing in females; modulated gut microbiota.	Supports gut–brain axis role in ASD.	[168]
ICR mouse (VPA model).	Probiotic fermented milk with *Lpb. plantarum* ST-III	6–8 weeks/ M and F.	Impaired locomotor behavior, increased anxiety, and deficient sociability. Altered gut microbiota diversity and composition.	*Lpb. plantarum* ST-III improved the impaired social interaction in male ASD mouse model and the autistic-like behaviors in male mice by modulating specific gut microbes.	Highlights effects on microbiota–gut–brain axis in ASD.	[195]
C57BL/6 WT mouse (MIA model).	Probiotic *Lpb. plantarum N*-1.	Adult/ M only.	Social interaction deficits; anxiety-like and depressive-like behavior; gut dysbiosis.	LPN-1 intervention improved autism-like behaviors in mice, including anxiety and depression, possibly via regulating the gut microbiota.	Supports microbiota–gut–brain axis role in ASD.	[196]
C57BL/6J mice (VPA model).	*Lpb. plantarum* PS128.	1 month/M and F; M only for behavioral tests.	ASD-like behaviors (social deficits, anxiety, cognitive impairments); reduced dendritic complexity, spine density, impaired synaptic signaling (Erk 1/2, PKA, CaMKIIα).	PS128 improved sociability, anxiety, and cognition in VPA mice; increased *Bifidobacterium* abundance; improved synaptic plasticity, dendritic structure, and glutamate receptor expression.	Supports gut–brain axis role in ASD.	[197]
Wistar rats (VPA model).	Prenatal probiotic treatment (*Lpb. plantarum* UBLP-40, *Lcb. casei-* UBLC-42, *L. acidophilus* UBLA-34, *L. bulgaricus L. bulgaricus* UBLB-38); postnatal prebiotic treatment (inulin).	PND 08–PND 50 /M and F.	ASD-like behaviors; impaired social interaction, memory deficits, repetitive behavior.	*Lactobacillus* strains have reversed autistic deficits and improved immune functions.	Supports gut–brain axis role in ASD.	[198]
Wistar rats (VPA model).	Probiotic VSL3# and prebiotic BTAGEN^®^.	PND22–PND63/M only.	VPA induced ASD-like behaviors. Differences in abundance of *Bacteroidetes*/*Firmicutes* ratio.	Probiotic and combined treatments improved autistic-like behaviors and the *Bacteroidetes*/*Firmicutes* ratio. Probiotic treatment decreased serum IL-6 levels and increased IL-10 levels.	Probiotic treatment shows translational potential.	[199]
Wistar rats (VPA model + antibiotics cocktail).	Probiotics *Lacticaseibacillus rhamnosus* GG, *Lpb. plantarum* 299v.	PND 2–21/M only.	VPA and chronic depletion of the gut microbiota induced ASD-like behavioral alterations.	The probiotic treatment was capable of re-establishing normal social behavior.	Probiotic treatment shows therapeutic potential.	[200]

^1^ TCS: triclosan, VPA: valproic acid, MIA: maternal immune activation, VSL3#: (Ferring Pharmaceuticals, Germany), containing eight different bacterial strains, namely *Streptococcus thermophilus* BT01, *Bifidobacterium breve* BB02, *Bifidobacterium animalis* subsp. lactis BL03, *Bifidobacterium animalis* subsp. lactis BL04, *Lactobacillus acidophilus* BA05, *Lactiplantibacillus plantarum* BP06, *Lacticaseibacillus paracasei* BP07, *Lactobacillus helveticus* BD08; BTAGEN^®^ (Klaire Labs, Reno, NV, USA), made of 70% inulin and the other prebiotic components were arabinogalactan.

**Table 3:** Clinical trials enrolling ASD children and adolescents involving a probiotic *Lpb. plantarum* intervention.

Study Design	Sample Size/Population	Age/Gender	Intervention (Strain, Dose, Duration)	Main Results	Conclusion/Clinical Relevance	Reference
Double-blind, randomized, parallel, placebo-controlled trial.	80 boys with ASD; 71 completed the study (PS128 *n* = 36, placebo *n* = 35); all in Taiwan.	Age 7–15 years, all males.	*Lpb. plantarum* PS128: 3 × 10^10^ CFU/capsule, 1 capsule/day, for 4 weeks.	PS128 group showed improvements in ABC-T (body/object use), SRS total, CBCL (anxiety, rule breaking), and in SNAP-IV.	PS128 may improve ADHD-like symptoms (hyperactivity/impulsivity) in younger children (age 7–12). Potential for age-specific psychobiotic intervention.	[146]
Randomized, double-blind, placebo-controlled, two-stage pilot trial.	35 individuals with ASD.	Age 3–20 years; 26 males/9 females.	*Lpb. plantarum* PS128 (6 × 10^10^ CFU/day, oral) for 16 weeks; from week 16 both groups received intranasal oxytocin; total duration 28 weeks.	Probiotic + oxytocin group showed greater improvement in CGI-I; trends toward improvement in ABC total, stereotypic behavior, and SRS cognition scores; enhanced gut microbiome connectivity and specific taxa correlations.	The combination of PS128 and oxytocin showed synergistic effects, suggesting potential for improved ASD core symptoms and gut–brain axis modulation. Combined therapy showed significant improvements to gut microbiome dysbiosis.	[208]
Retrospective observational study.	131 autistic children and adolescents.	Aged 45–127 months (mean age 7.9 years), males and females.	*Lpb. plantarum* PS128, 3 × 10^10^ CFUs (weight less than 30 kg) and 6 × 10^10^ CFUs (weight higher than 30 kg). Duration 6 months.	Significant improvements observed in CGI scores after PS128 administration; effects were more pronounced in participants with gastrointestinal symptoms.	Supplementation with *Lpb. plantarum* PS128 was associated with behavioral improvements in children and adolescents with ASD, particularly those with comorbid GI symptoms.	[207]
Double-blind, randomized, placebo-controlled clinical trial.	35 individuals with ASD.	Age 3–25, 26 males/9 females.	*Lpb. plantarum* PS128, 6 × 10^10^ CFU/day for 16 weeks.	Probiotic group showed specific improvements in behavior, inflammatory markers, gut microbiota diversity.	PS128 may be beneficial in subgroups of ASD children, particularly those with specific autoimmune/inflammatory profiles.	[209]
Double-blind, randomized, parallel, placebo-controlled trial.	*n* = 82 randomized, 79 completed.	Male and female children, aged 2.5–7 years, with ASD.	*Lpb. plantarum* PS128, 6 × 10^10^ CFU/day, oral. Duration of 4 months in two stages of interventions, each lasting 2 months.	PS128 significantly improved ASEBA * anxious/depressed scores and ADHDT hyperactivity; some GI symptoms improved in both groups after PS128 phase.	PS128 may reduce anxiety and hyperactivity symptoms in young children with ASD; some gastro-intestinal benefits observed.	[210]
Randomized, double-blind, placebo-controlled parallel-group trial.	46 preschoolers with ASD.	Age 18–72 months; 35 males/11 Females.	Probiotic Vivomixx^®^; daily dose (900 billion CFU first month, 450 billion CFU last 5 months); 6 months.	EEG changes correlated with behavioral and inflammatory measures.	Probiotics induced EEG changes resembling typical brain activity; suggest neuroplastic effects and gut–brain modulation in ASD.	[179,211]
Randomized, double-blind, placebo-controlled crossover pilot trial.	13 children with ASD (10 completed).	Age 3–12 years, males and females.	Visbiome^®^ probiotic; 9 × 10^11^ CFU/day; 19 weeks × 2 phases.	Improvement in GI symptoms (PedsQL GI module); reduced parent-reported target GI symptoms.	The Visbiome^®^ formulation suggested a health benefit in children with ASD and GI symptoms who retained Lactobacillus.	[212]
Double-blind, randomized, parallel, factorial, placebo-controlled trial.	85 children with; no GI symptoms group (NGI), GI symptoms group (GI); all Italian; 63 children completed the trial.	ASD children aged 1.5–6 years, 71 males (84%).	Probiotic DSF (Vivomixx^®^/Visbiome^®^), 2 packets/day (month 1), 1 packet/day (months 2–6). Duration of 6 months.	In NGI subgroup: significant reduction in ADOS-CSS. In GI subgroup: probiotics improved GI symptoms, adaptive functioning, and multisensory processing.	Probiotics improved social affect scores in children without GI issues, and enhanced GI sensory, and adaptive functions in those with GI symptoms.	[213]

* ASEBA: Achenbach System of Empirically Based Assessment; ADHDT: Attention-Deficit/Hyperactivity Disorder Test; ADOS-CSS: Autism Diagnostic Observation Schedule–Calibrated Severity Score; CGI-I: Clinical Global Impression scale; ABC: Aberrant Behavior Checklist; SRS: Social Responsiveness Scale; ABC-T: Autism Behavior Checklist–Taiwan version; CBCL: Child Behavior Checklist; SNAP-IV: Swanson, Nolan, and Pelham-IV; PedsQL: Pediatric Quality of Life Inventory; Probiotic DSF (Vivomixx^®^/Visbiome^®^): DSF, a patented mixture already approved for use in children (marketed as Vivomixx^®^ in EU, Visbiome^®^ in USA), each packet contained 450 billions of eight probiotic strains, namely *Streptococcus thermophilus*, *Bifidobacterium breve*, *Bifidobacterium longum*, *Bifidobacterium infantis*, *Lactobacillus acidophilus*, *Lactiplantibacillus plantarum*, *Lacticaseibacillus paracasei*, *Lactobacillus delbrueckii* subsp. *bulgaricus.*; EEG: electroencephalography.
